# Identification of PARP12 Inhibitors By Virtual Screening and Molecular Dynamics Simulations

**DOI:** 10.3389/fphar.2022.847499

**Published:** 2022-08-09

**Authors:** Tahani M. Almeleebia, Shahzaib Ahamad, Irfan Ahmad, Ahmad Alshehri, Ali G. Alkhathami, Mohammad Y. Alshahrani, Mohammed A. Asiri, Amir Saeed, Jamshaid Ahmad Siddiqui, Dharmendra K. Yadav, Mohd Saeed

**Affiliations:** ^1^ Department of Clinical Pharmacy, College of Pharmacy, King Khalid University, Abha, Saudi Arabia; ^2^ School of Biotechnology, IFTM University, Moradabad, India; ^3^ Department of Clinical Laboratory Sciences, College of Applied Medical Sciences, King Khalid University, Abha, Saudi Arabia; ^4^ College of Applied Medical Sciences, Najran University, Najran, Saudi Arabia; ^5^ Department of Clinical Laboratory Sciences, College of Applied Medical Sciences, University of Hail, Hail, Saudi Arabia; ^6^ Department of Medical Microbiology, Faculty of Medical Laboratory Sciences, University of Medical Sciences and Technology, Khartoum, Sudan; ^7^ Department of Transplant Immunology and Immunogenetics, All India Institute of Medical Sciences, New Delhi, India; ^8^ Department of Pharmacy, Gachon Institute of Pharmaceutical Sciences, College of Pharmacy, Gachon University of Medicine and Science, Incheon, South Korea; ^9^ Department of Biology, College of Sciences, University of Hail, Hail, Saudi Arabia

**Keywords:** PARP12, ZINC-FDA, virtual screening, MMGBSA, MD simulations

## Abstract

Poly [adenosine diphosphate (ADP)-ribose] polymerases (PARPs) are members of a family of 17 enzymes that performs several fundamental cellular processes. Aberrant activity (mutation) in PARP12 has been linked to various diseases including inflammation, cardiovascular disease, and cancer. Herein, a large library of compounds (ZINC-FDA database) has been screened virtually to identify potential PARP12 inhibitor(s). The best compounds were selected on the basis of binding affinity scores and poses. Molecular dynamics (MD) simulation and binding free energy calculation (MMGBSA) were carried out to delineate the stability and dynamics of the resulting complexes. To this end, root means deviations, relative fluctuation, atomic gyration, compactness, covariance, residue-residue contact map, and free energy landscapes were studied. These studies have revealed that compounds ZINC03830332, ZINC03830554, and ZINC03831186 are promising agents against mutated PARP12.

## 1 Introduction

Poly [adenosine diphosphate (ADP)-ribose] polymerases (PARPs) are the nuclear enzymes that regulate fundamental cellular processes involving protein degradation and gene expression (Kim et al., 2011; Bai and Virag, 2012; Langelier et al., 2018). PARPs use nicotinamide adenine dinucleotide (NAD+) for the process of post-translation that modify substrate proteins with ADP-ribose, a vital process referred to as ADP ribosylation (Carter-O'Connell and Cohen, 2015; Griffiths et al., 2020). The majority of PARP family members are monoPARPs as they catalyze the transfer of mono-ADP-ribose (MAR) onto their substrates (MARylation) (Cohen, 2020). In contrast, polyPARPs attach polymers of poly-ADP-ribose (PAR) onto their substrates (PARylation) (Carter-O'Connell and Cohen, 2015; Lu et al., 2019). PARP1 is a well-known cancer target involved in DNA damage-induced cellular stress/genetic mutation/cytotoxic chemotherapy (Gozgit et al., 2021). There are four drugs readily available on the market, and other drugs are at their late-stage development (Schiewer et al., 2012; Schiewer and Knudsen, 2014; Green et al., 2015; Rudolph et al., 2021). PARP12 is also a mono-ART of the PARP family, which controls the regulation of cell survival and regrowth (Catara et al., 2017). The function of PARP12 is the mono-ADP-ribosyltransferase that mediates mono-ADP-ribosylation of target proteins, although the molecular mechanism responsible for cell survival is still unclear (Catara et al., 2017; Grimaldi et al., 2020). Recently, PARP12 involvement in intracellular membrane transport at this point deserves much attention and alternative effective strategies (Gozgit et al., 2021). Several studies also suggested that PARP12 is a novel target that reduces breast cancer resistance to genotoxic stress (Ke et al., 2019). Thus, PARP12 is marked as a potential target that must be utilized further to identify and design potential inhibitors/vaccine candidates at the molecular and genetic levels. The authors identify PARP1 potent targets and various computational studies that have been published to utilize the ZINC-FDA library and traditional Chinese medicine (Costantino et al., 2001; Chen et al., 2014; Maksimainen et al., 2021; Sahin and Durdagi, 2021). Similarly, there are other recently published theoretical studies on PARP2 to find novel benzimidazole derivatives (Venugopal and Chakraborty, 2021).

Bioinformatics tools and databases are wonderful approaches to identify the novel drugs and vaccine candidates as an alternative strategy. Taking the lead from here, the current study aimed to utilize the available small-molecule ZINC database, which is a publicly available and accessible database. We used a comprehensive *in silico* approach involving literature mining, virtual screening based molecular docking, pharmacological analysis, and large-scale molecular dynamics (MD) simulations to identify potential PARP12 inhibitors from a pool of ZINC-FDA. We analyzed 3,100 chemically diverse pharmacologically active compounds (Sterling and Irwin, 2015). Based on the comparative analysis of docking scores, binding affinity, and energies, we selected four compounds, namely, ZINC03830332, ZINC03830554, ZINC03831186, and ZINC03831189. Consequently, the compounds were analyzed for ADME/T properties and were potential drug-like candidates. Furthermore, we analyzed the conformational stability of the docked complexes using MD simulations with the help of various parameters such as root mean square deviation (RMSD), root mean square fluctuation (RMSF), radius of gyration (Rg), solvent accessible surface area (SASA), free energy landscapes (FEL), H-bond monitoring, principal component analysis (PCA), and density distribution map (Ahamad et al., 2021b). Based on MD simulation results, two compounds (ZINC03830332 and ZINC03831186) were identified as PARP12 inhibitors.

## 2 Materials and Methods

**FIGURE 1:**
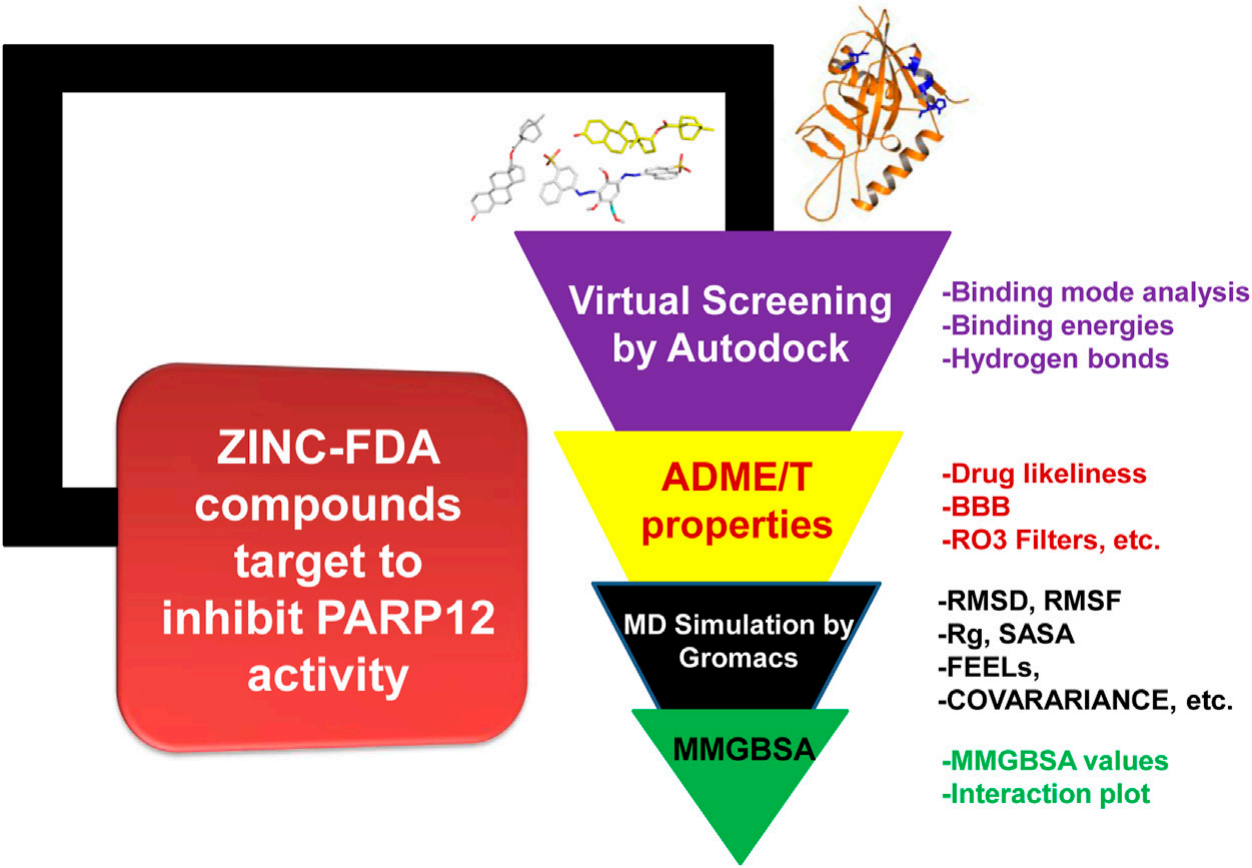
Optimized protocol employed in this work.

The work was carried out on a Dell workstation, 256 GB RAM, and NVIDIA graphics card GeForce GTX 1080Ti GDDR5 11 GB. The workstation was installed with several bioinformatics software applications such as GROningen MAchine for Chemical Simulations (GROMACS), XMGrace (GROMACS results analysis), and Schrödinger Molecular Mechanics Generalized Born Surface Area (MMGBSA) and 2D plots. The detailed workflow used in this study is represented in Figure 1.

### 2.1 Protein Preparation and Grid Generation for Docking Studies

The human PARP12 with four-point mutations (Val570, Gly573, His577, and Phe607) has been selected in this study. The PARP12 has a 701 amino acid length, and the crystal structure (PDB = 6V3W) is available with the PARP catalytic domain (496–680 amino acids). To identify the potential surface structural pockets, their shape and volume, internal cavities of the protein, and surface areas, 6V3W was given as the input (Gozgit et al., 2021). The active site and the interactive residues were identified using the online tools CASTp and PDBsum and verified using the literature (Laskowski, 2001; Dundas et al., 2006; Gozgit et al., 2021). The ligands (FDA-ZINC compounds) were prepared using AutoDock tools (ADT) by converting the PDB file into a PDBQT format (Huey and Morris, 2008; Ahamad et al., 2019). The crystal structure of PARP12 was prepared by using the ADT protein preparation wizard. Polar hydrogen (H) bonds and missing H-atoms were added while the water molecules and heteroatoms were deleted from the 3D structure. Energy minimization was performed with a default constraint of 0.3 Å root mean square (RMS), and charges were assigned. Finally, the clean structure was saved as a PDBQT file, and docking was performed by AutoDockVina (version 1.1.2) software (Trott and Olson, 2010). The size of the grid box selected was 106 Å × 108 Å × 106 Å, respectively, and generated around the centroid of the compounds and PARP12 complex. Default parameter settings were used during docking. The 3D structure of the docked complexes was analyzed with the help of the PyMOL visualization tool (DeLano, 2002).

### 2.2 Structure-Based Virtual Screening

The PDBQT formatted ligands (ZINC-FDA compounds) were screened against PARP12. A total of 3,100 molecules were retrieved from the ZINC-FDA database to identify potential PARP12 inhibitors (Sterling and Irwin, 2015). The compounds were ranked on the basis of their binding energy scores and docking interaction poses, and the top four compounds were selected.

### 2.3 Molecular Dynamics (MD) Simulations

MD simulations were carried out for the best-docked complexes using GROMACS (version 5.18.3) to determine the PARP12 enzyme behavior in the presence of water (Van Der Spoel et al., 2005; Abraham et al., 2015). The topology of PARP12 was created *via* the GROMOS9643a1 force field (Pronk et al., 2013). The PRODRG server was used to generate molecular topologies and coordinate files (Schuttelkopf and van Aalten, 2004). All the systems were solvated using a simple point-charge model (SPC/E) in a cubic box (Mark and Nilsson, 2001). To neutralize the system, 0.15 M counter ions (Na^+^ and Cl^−^) were added. The energy minimization of all the neutralized systems was performed using the steepest descent and conjugate gradients (50,000 steps for each). The volume (NVT) regulation and pressure (NPT) were run for system equilibration. Steepest descent followed by conjugate gradient algorithms was utilized on enzyme-ligand complexes. The NVT ensemble at a constant temperature of 300 K and the constant pressure of 1 bar was employed. The SHAKE algorithm was used to confine the H-atoms at their equilibrium distances and periodic boundary conditions.

Moreover, the particle mesh Ewald (PME) method was used to define long-range electrostatic forces (Darden et al., 1993; Lee et al., 2016; Wang et al., 2016). The cut-offs for van der Waals and Columbic interactions were 1.0 nm. The LINC algorithm was used to constrain the bonds and angles (Hess et al., 1997). Finally, the production runs were performed for the period of 500 ns The energy, velocity, and trajectory were updated at the time interval of 10 ps The MD simulation analyses were performed, and trajectoris were found by GROMACS utilities and MDTraj-based Python scripts (McGibbon et al., 2015). The Cα-atom deviations of PARP12 and complexes were utilized to calculate using RMSD and RMSF for relative fluctuations of each amino acid. The compactness was measured by Rg, while the SASA was used to know the electrostatic contributions of molecular solvation. PCA is one of the best techniques that help reduce complexity to extract the intensive motions in MD simulation analysis. The matrix was formed for the MD trajectory after excluding rotational and translational movements. So the essential dynamics protocol was implemented to calculate the eigenvectors and eigenvalues and their projections along with the first two principal components (PCs). The diagonalized covariance matrix, eigenvectors, and eigenvalues were identified. The eigenvector of the matrix gives the multidimensional space and the displacement of atoms in the molecule in each direction. This analysis processed the essential subspace built on knowing each atom’s movements, which were plotted by Cartesian trajectory coordinates using GROMACS utilities (Ahamad et al., 2021a).

### 2.4 MMGBSA Calculation

The MMGBSA (ΔG_bind_) was calculated by the Schrödinger Prime module to compute the ligand binding energies. The prime-MMGBSA was used for rescoring the docked poses.

ΔG is calculated by the following equation:

ΔGbind = E_complex (minimized) − E_ligand(minimized)− E_receptor (minimized),

where ΔG_bind_ specifies the binding free energy, E__complex_ is the input for the energy minimization of the protein-ligand complexes, E__receptor_ for the free protein, and E__ligand_ for free ligands. The sum of molecular mechanical minimized energies including van der Waals interaction, internal energies, electrostatic energies, and solvation free energies were also calculated for the docked conformations.

## 3 Results

Despite the fact that PARP12 is an intriguing target for treating various ailments, no computational studies on PARP12 have been carried out yet. Based on this notion, we screened 3,100 biologically active molecules from the FDA-approved drug databas**e** to find out lead(s) against the PARP12 enzyme. Out of these, four compounds (ZINC03830332, ZINC03830554, ZINC03831186, and ZINC03831189, Supplementary Figure S1) were selected on the basis of their docking score and interaction with the receptor. Moreover, the four best hits were analyzed for Lipinski and ADME/T properties. All-atom MD simulations were further performed on the mutant PARP12 and four-drug complexes. The results of the studies are discussed herein.

### 3.1 Molecular Docking

**FIGURE 2:**
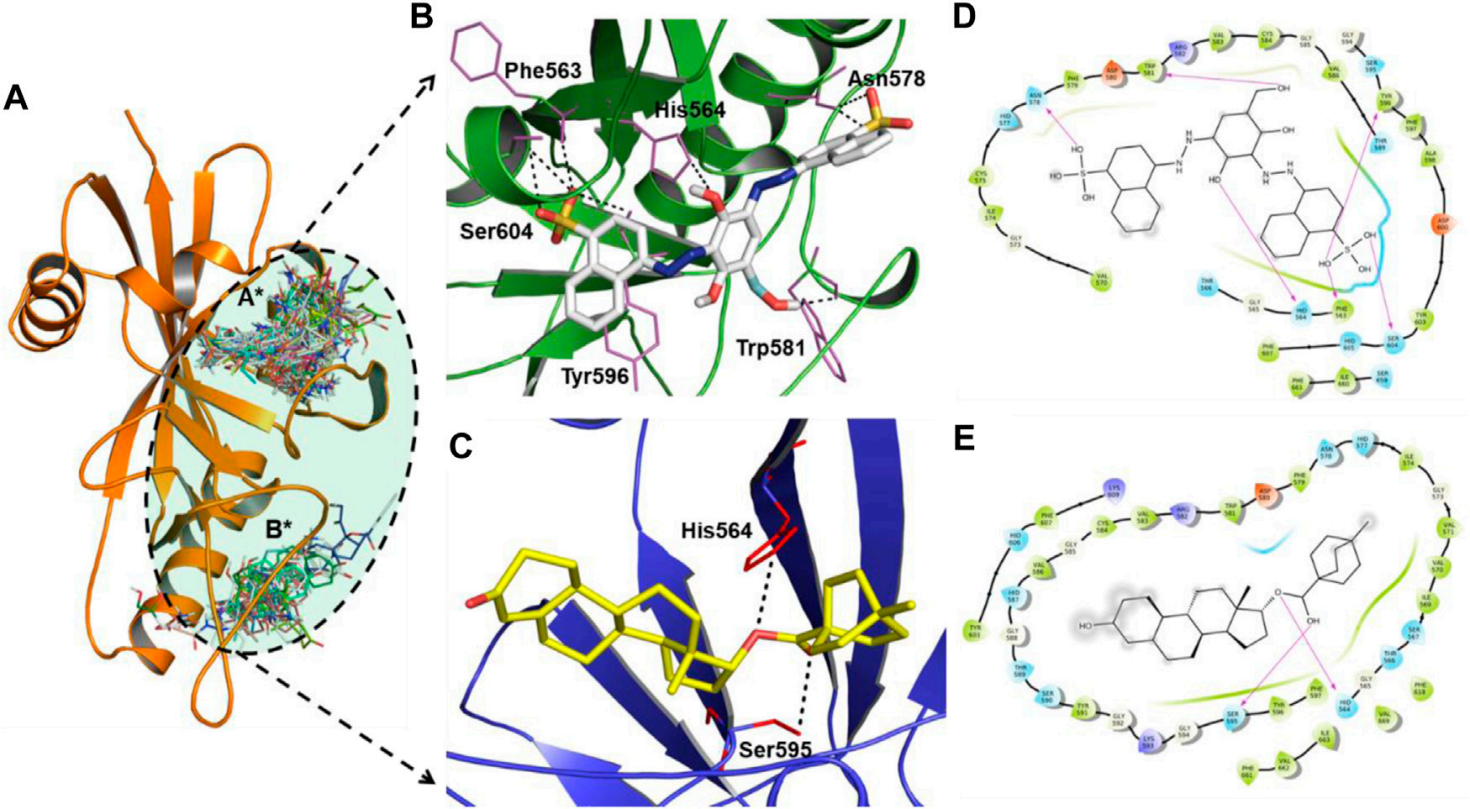
**(A)** Close-up view of the binding pockets (A* and B* encircled by black dotted lines) of the PARP12 enzyme. H-bond interactions between **(B)** ZINC03830332 (white stick), **(C)** ZINC03831186 (yellow stick), and PARP12 receptor (in green and blue color cartoon models). **(D,E)** 2D plot of the complexes.

Molecular docking is a powerful tool for lead discovery and to underpin molecular interactions. Figure 2A depicts the binding pockets (A* and B* encircled by black dotted lines) of the PARP12 enzyme and binding poses of the docked ligands. Herein, we have shortlisted four compounds that carried the best binding free energies, as well as polar and non-polar interactions. Molecular docking allows prediction of the preferred pose and binding orientations of a ligand inside a receptor binding site corresponding to the best energy score. The top-scoring shortlisted four compounds were docked in the A* binding pocket. However, B* binding pocket interaction compounds have fewer binding energies. Figures 2B,C and Supplementary Figure S1 show the molecular interaction between the four hits and receptor. Table 1 collects the results of the molecular docking study of four shortlisted ZINC-FDA compounds. It is clear from the figures that ligands interacted with the receptor via various polar and non-polar modes (Figures 2D,E and Supplementary Figure S1). It can be seen that ZINC03830332 formed six H-bonds with Phe563, His564, Asn578, Trp581, Tyr596, and Ser604 residues of the receptor with a docking score of −12.40 kcal/mol. On the other hand, the docking score for the PARP12-ZINC03831186 complex was −11.40 kcal/mol which formed two H-bonds with the receptors His564 and Ser595. The two other ligands ZINC03830554 and ZINC03831189 exhibited relatively higher docking scores (−11.80 kcal/mol and −12.11 kcal/mol, respectively) and interacted *via* seven (Phe563, His564, His577, Asp580, Tyr596, Ser604, and Lys666) and two (Gly594 and Ser604) H-bonds. The 2D plot analyses of PARP12-ligand complexes revealed that the complexes were also stabilized through hydrophobic interactions from nearby residues.

**TABLE 1:** Average RMSD, Rg, SASA, and MMGBSA values of the mutant receptor and complexes. Molecular docking results of four shortlisted ZINC-FDA compounds.

Mutant system and complex	RMSD (nm)	Rg (nm)	SASA (nm^2^)	MMGBSA ∆G score (kcal/mol)	Binding score (kcal/mol)	Residues involved in H-bonding	Residues involved in hydrophobic interaction
PARP12	0.43	1.57	101.70	-	-	-	-
ZINC03830332	0.32	1.59	102.17	−135.11	−12.40	Phe563, His564, Asn578, Trp581, Tyr596, and Ser604	Thr566, Val570, Gly573, Ile574, Cys575, His577, Phe579, Asp580, Arg582, Val583, Cys584, Gly585, Val586, Thr589, Gly594, Ser595, Phe597, Ala598, Asp600, Tyr603, His605, Phe607, Ser659, Ile660, and Phe661
ZINC03830554	0.34	1.58	104.02	−39.22	−11.80	Phe563, His564, His577, Asp580, Tyr596, Ser604, and Lys666	Val570, Gly573, Ile574, Val583, Gly565, Phe597, Ala598, Tyr603, and His606
ZINC03831186	0.32	1.59	103.26	−57.63	−11.40	His564 and Ser595	Gly565, Thr566, Ser567, Ile569, Val570, Val571, Gly573, Ile574, His577, Asn578, Phe579, Asp580, Trp581, Arg582, Val583, Cys584, Gly585, Val586, His587, Gly588, Thr589, Gly594, Ser595, Phe597, Ser590, Tyr591, Gly592, Lys593, Gly594, Phe597, Tyr603, His606, Phe607, Lys609, Phe661, Valy662, and Ile663
ZINC03831189	0.37	1.58	103.24	−51.92	−12.11	Gly594 and Ser604	Phe563, His564, Gly565, Thr566, Asn578, Phe579, Asp580, Trp581, Arg582, Val583, Cys584, Gly585, Val586, His587, Thr589, Gly592, Lys593, Ser595, Tyr596 Phe597, Ser590, Tyr591, Gly592, Ala598, Asp600, Ala601, Tyr603, His605, and Phe607

### 3.2 Physicochemical and ADME/T Studies

The shortlisted best four hits, namely, ZINC03830332, ZINC03830554, ZINC03831186, and ZINC03831189 compounds were used to calculate the pharmacological properties such as MW, dipole, SASA, FOSA, FISA, PISA, WPSA, volume, DonorHB, and AccptHB. We found that all the four molecules obeyed the rule of three (Ro3) (Kö;ster et al., 2011). Preferably, an orally active drug/compound should not cross more than one violation, but various exceptions are available (Lipinski, 2004). Two compounds (ZINC03830332 and ZINC03830554) violated one rule increase in MW, which is still acceptable. The results of the RO3 study revealed that the molecular weight of the compound was in the range of 422–693, with 4–10 H-bond acceptor and 0–14 H-bond donating units. The *in silico* absorption, distribution, metabolism, excretion (ADME), and toxicity (T) are the most widely used techniques in rational drug discovery as it gives a fair idea about the drug candidateship. In this context, we investigated the ADME/T and RO3 properties of the best four hits, and the results are compiled in Supplementary Table S1. The QikProp module output data generated for the compounds indicated that the blood–brain barrier (BBB) was in the range of −0.38 to −4.28 with QPlogPo/w between −0.55 and 5.73, QPlogKhsa between −0.63 and 1.14, SASA between 714.08 and 1,083.70, and log *K*p value ranging from −2.41 to −10.90. Overall, the four hits showed fair enough properties and ability needed for a drug to possess (Lee et al., 2003; Lipinski, 2016; Nazarbahjat et al., 2016; Cordeiro and Kachroo, 2020).

### 3.3 Molecular Dynamics Simulation Analysis

MD simulations have the ability to uncover various dynamic interactions between a ligand and receptor, their interaction mechanism, and stability. MD simulation was carried out at the 0 to 500 nanosecond (ns) time scale for each system with a total of 2,500 ns. The MD simulations of the mutant systems of PARP12 and ZINC03830332, ZINC03830554, ZINC03831186, and ZINC03831189 complexes were utilized to measure the structural changes and parameters such as RMSD, RMSF, Rg, SASA, H-bonds, and Gibbs free energy, i.e., PCA calculation, covariance matrix, and density distribution map. The results of the findings are discussed as follows.

#### 3.3.1 RMS Deviation and Principal Component Analysis

**FIGURE 3:**
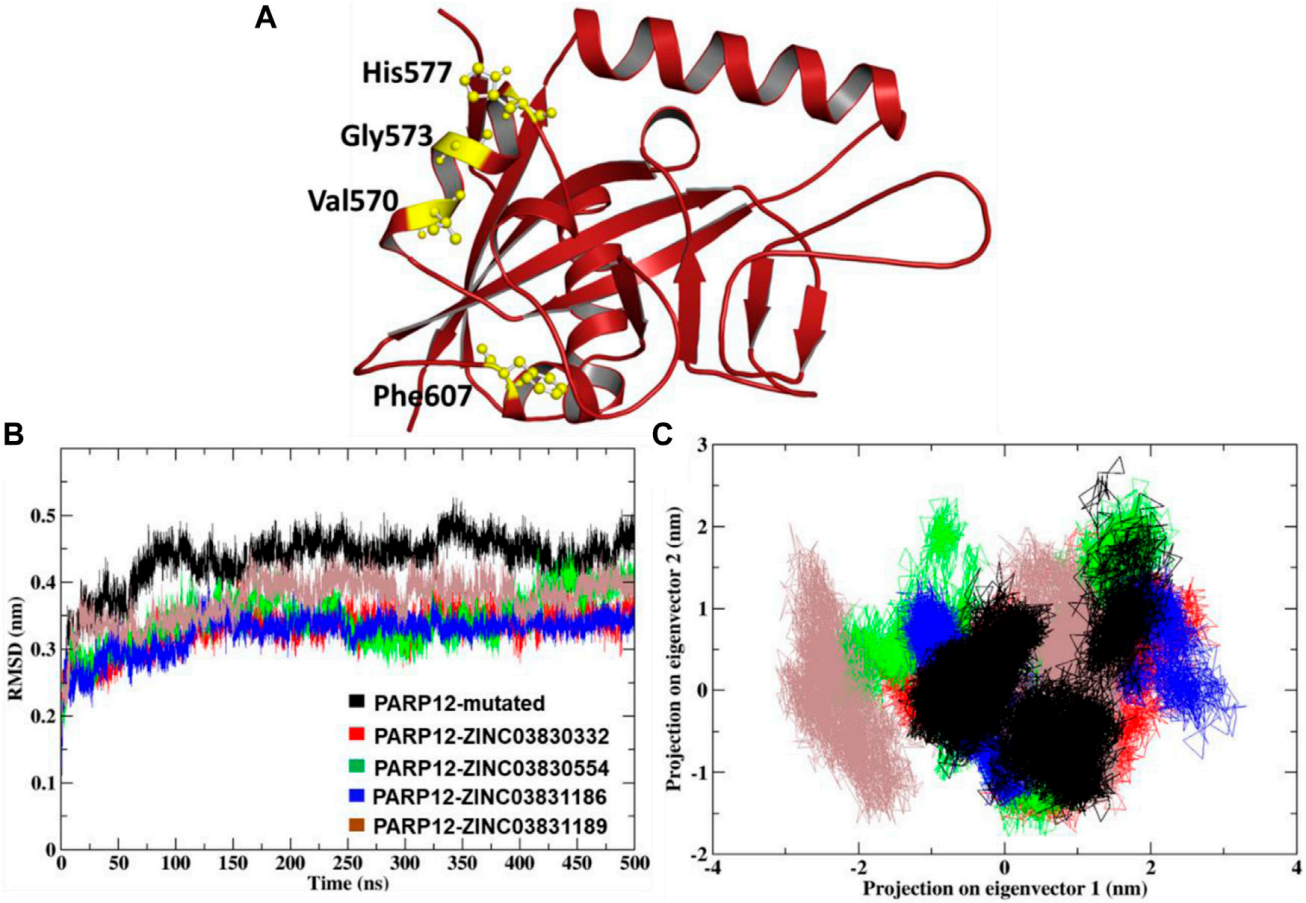
**(A)** Mutation induced by PARP12 structures, **(B)** comparison of the RMSD plot, and **(C)** dynamic motion of projection in the eigenvector 1 vs. 2 and the plot two generated for the MD complexes showing conformational space of Cα-atoms.

The MD simulation study result dictates the multidimensional data’s relative distance to reduce the dimensional space. Atom-positional RMSD value C-alpha (C-α) atoms were calculated for the mutant system and complexes (Figure 3A). The MD simulation of mutant PARP12 systems and complexes with ZINC03830332, ZINC03830554, ZINC03831186, and ZINC03831189 compounds provided average RMSD values of ∼0.43 nm, ∼0.32 nm, ∼0.34 nm, ∼0.32 nm, and ∼0.37 nm, respectively (Figure 3B). On the other hand, the mutant system of PARP12 attained equilibrium in 75 ns with a relatively higher average RMSD value due to the mutations in the helix and loop region. However, in the case of complexes, almost all the systems attained equilibrium within 30 ns and the steady-state in all the complexes except ZINC03831189, which possess a slightly greater RMSD value (∼ 0.37 nm). Overall, the complexes involving ZINC03830332, ZINC03831186, and ZINC03830554 possessed a stable binding with PARP12.

The 2D projection of trajectories on eigenvectors 1 and 2 is the part of essential dynamics (ED) and reflect an overall expansion of the structural dynamics. The large-scale average motion indicated higher atomic fluctuations, and its flexibility measures the atomic mobility of the mutant and complexes. The eigenvector values of the docked complexes ZINC03830332, ZINC03830554, ZINC03831186, and ZINC03831189 were calculated. We found that the essential subspace for the mutant PARP12 of eigenvector 1 in the range from −1.5 to −2.5 nm, vs. eigenvector 2 in the range from −1.5 to 3 nm of a larger cluster for the mutant PARP12 (Figure 3C). Similarly, we extensively investigated the global motion for the complex of the ZINC03830332 compound. We found that the eigenvector 1 in the range from −1.5 nm to −2 nm vs. eigenvector 2 in the range from −1 to 1.5 nm occupied the smallest subspace throughout the MD simulation. However, compounds ZINC03830554 and ZINC03831186 had eigenvector 1 in the range −2 to 2 nm vs. eigenvector 2 in the range −1.5 to 2.5 nm and eigenvector 1 in the range −1 to 3 nm vs. eigenvector 2 in the range −1 to 1 nm, respectively. The compound ZINC03831189 had eigenvector 1 in the range −3 to 1.5 nm vs. eigenvector 2 in the range −1.5 to 2 nm. Out of four complexes, we found a more restricted subspace that leads to well-defined internal motion with ZINC03830332, ZINC03830554, and ZINC03831186 complexes.

**FIGURE 4:**
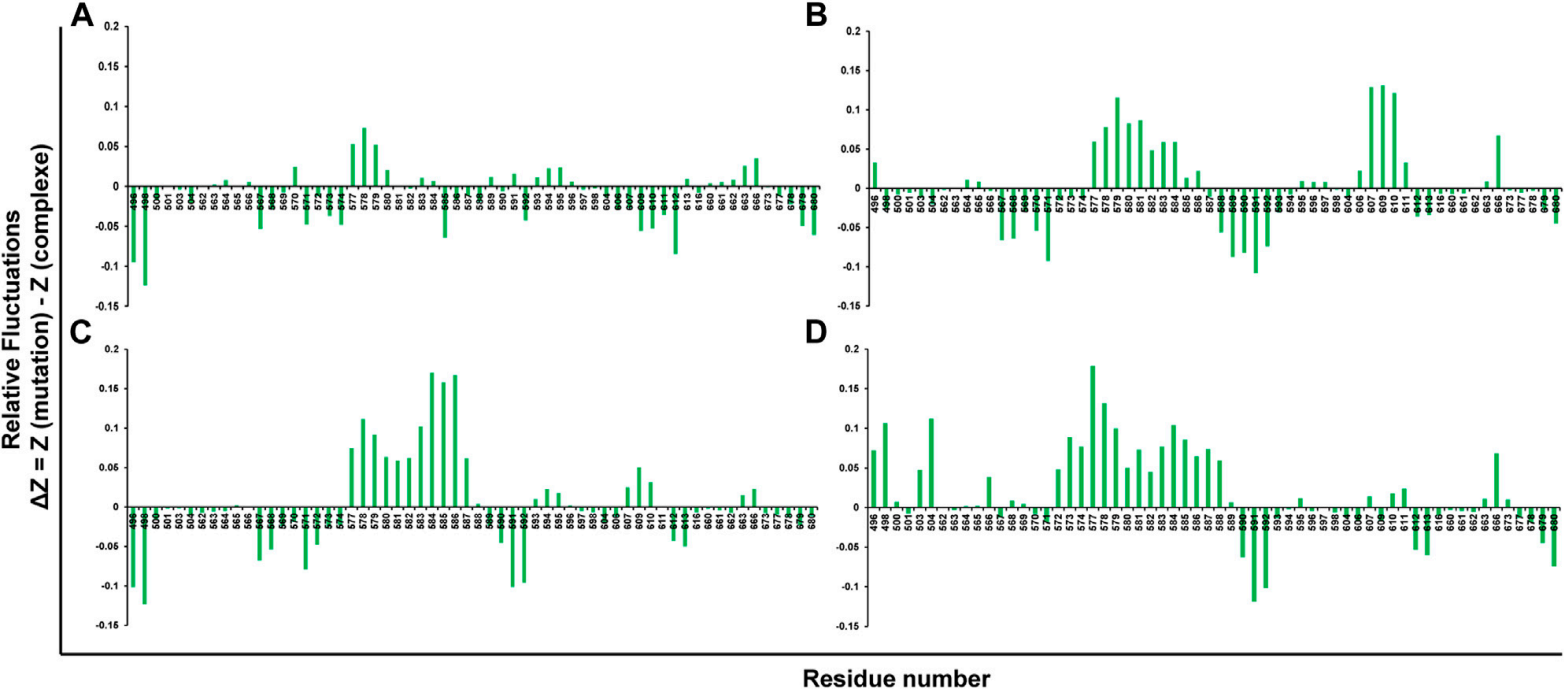
Root mean square fluctuations (RMSFs) of mutant systems of PARP12 and **(A)** ZINC03830332, **(B)** ZINC03830554, **(C)** ZINC03831186, and **(D)** ZINC03831189.

The rigid regions and flexibility could be identified by RMSF analysis. The plot’s negative drift indicates the increased movement of the Ca atoms of relative fluctuations. It was found that the compound ZINC03830332 showed stable positive amino acid fluctuations at F563-T566, V570, H577-W581, V583-596, Q613, I660-I663, K666, and Y673. A similar trend was found for the other two compounds with a high degree of flexibility (Figures 4A–D).

**FIGURE 5:**
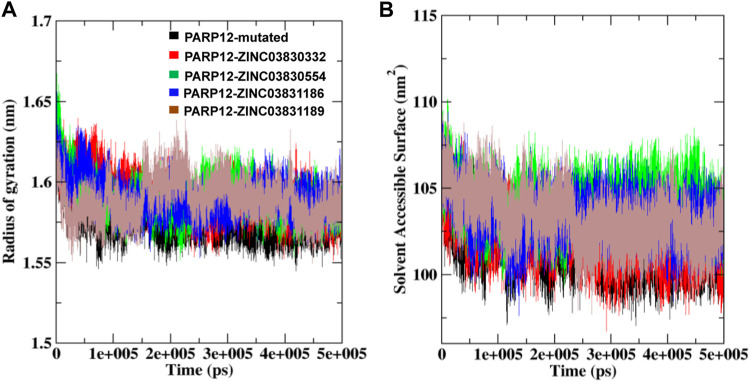
**(A)** Rg and **(B)** SASA fluctuations per residue variation plot analysis of the mutant system and in the presence of compounds with PARP12 at total simulation time.

To gain more insight into the flexibility, the Rg plot, which measures the mass-weighted RMS distance of a cluster of atoms from their center of mass, was determined. The Rg analysis of C-α atoms of ligand–receptor docked complexes average values was calculated between 0 and 500 ns. Figure 5A illustrates the Rg plot, and the results indicated a compact conformation of the complexes, as compared to the native enzyme. For instance, average values ∼1.57 nm, ∼1.59 nm, ∼1.58 nm, ∼1.59 nm, and ∼1.58 nm were found for PARP12 and ZINC03830332, ZINC03830554, ZINC03831186, and ZINC03831189 complexes, respectively (Table 1). Overall, the result suggested mutant expanded conformation while compact conformation for the complexes.

To understand the behavior of the PARP12 mutant system and all the complexes, we also performed a SASA analysis which revealed the PARP12 surface that can be accessed in the solvent (water). The average SASA value of the mutant PARP12, complexes with ZINC03830332, ZINC03830554, ZINC03831186, and ZINC03831189 were found to be 101.70, 102.17, 104.02, 103.26, and 103.24 nm^2^, respectively, (Figure 5B and Table 1).

#### 3.3.2 Hydrogen Bond Vetting

Hydrogen bonding plays a significant role in knowing the complexes’ stability. To determine the binding affinity of the selected compounds toward the PARP12 receptor, MD trajectories were analyzed, and an H-bond investigation was carried out to determine the total number of bond formations. The ZINC03830332 and ZINC03830554 formed 0–14 H-bonds consistent for both the compounds’ complexes with PARP12 throughout the 500 ns MD simulation. Similarly, the compound ZINC03831186 complex has quite less 0–5 number of H-bonds, whereas ZINC03831189 docked complexes showed H-bonds 0–4 (supplementary Figures S3A–D). The intermolecular H-bond examination revealed that the complex containing ZINC03830332 possessed a minimum of fourteen H-bonds during the MD simulation. The number of H-bond interactions also increased in the case of ZINC03830554 during the simulation. Contrarily, complexes containing ZINC03831186 and ZINC03831189 were unable to increase the number of H-bonds; however, both compounds showed consistency in the H-bonds’ profile.

#### 3.3.3 Free Energy Landscape Analysis

**FIGURE 6:**
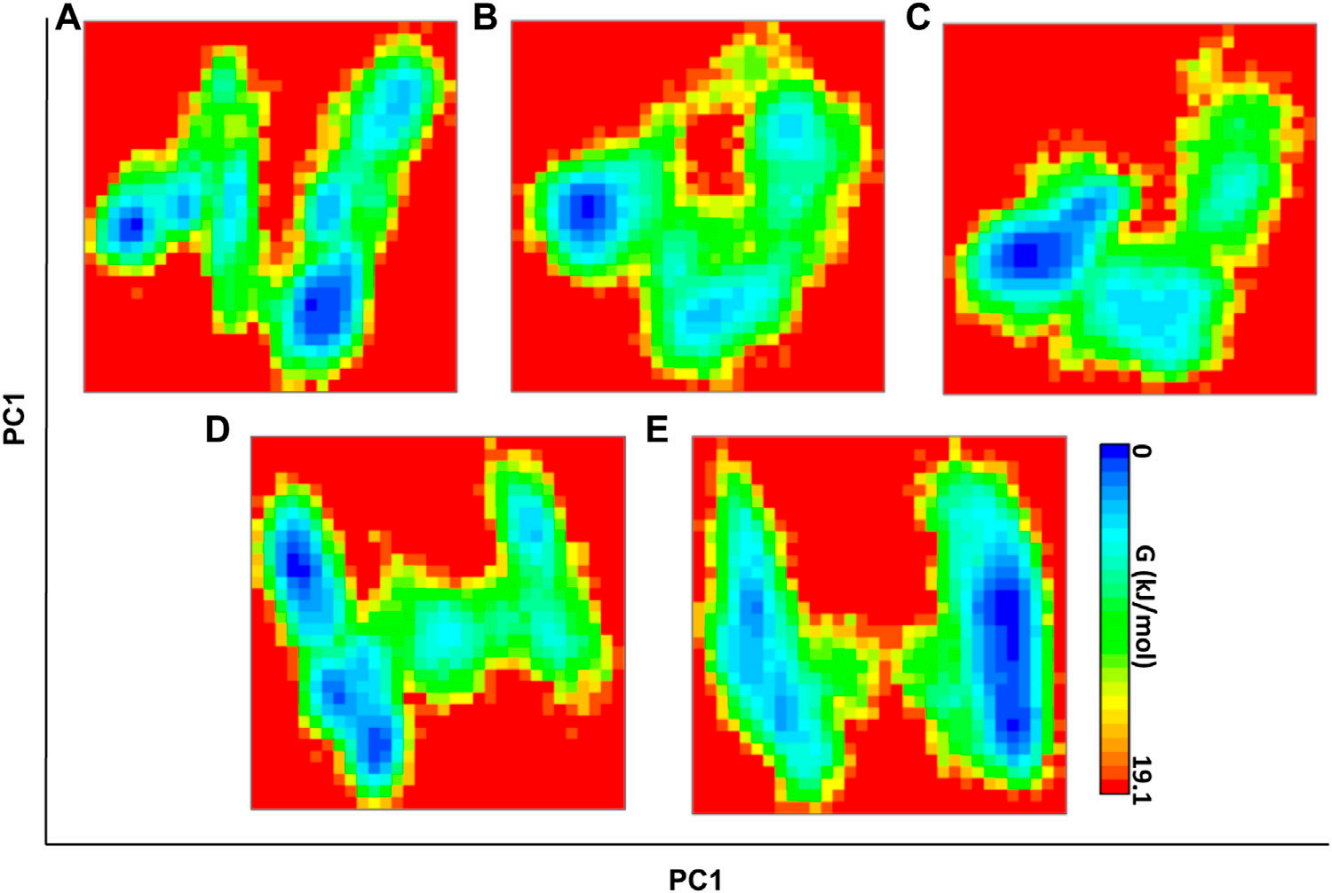
FEL direction of motion and magnitude analysis of the mutant PARP12 **(A)** and complexes with ZINC03830332 **(B)**, ZINC03830554 **(C)**, ZINC03831186, and **(D)** ZINC03831189 **(E)** throughout MD simulations. The color bar denotes the relative free energy value between 0 and 19.1 kcal mol^−1^.

To monitor the distinct conformation of binding, FEL analysis was performed. The principal components (PCs) dictate the most dominant internal modes of motion of a corresponding system. In the ligand binding or complex formation in MD, through PCA, we found the most probable and dominant conformation changes of the protein during binding. The first PC1 and second PC2 utilized projected eigenvectors generated based on PCA (Stein et al., 2006). The contour map of the FEL derived conformational changes of the mutant system and complexes of PC1 and PC2 eigenvectors (Figures 6A–E). The Gibbs free energy landscape shows the global energy minima states (Ali et al., 2019; Ahamad et al., 2021b). The energetically favored PARP12 complex conformation is indicated by dark blue spots, while the unfavorable conformations are indicated by yellow spots. The values of FEL ranged from 0 kJ/mol to 19.1 kJ/mol, 18.2 kJ/mol, 18.2 kJ/mol, 18.1 kJ/mol, and 16.7 kJ/mol for the mutant PARP12 and ZINC03830332, ZINC03831186, ZINC03831189, and ZINC03830554 docked complexes, respectively. The FEL results demonstrated several well-defined energy minima, corresponding to the metastable conformational states. From Figure 6, it is clear that the main free energy wells in the global free energy minima area were altered when selected ZINC-FDA compounds were bound with PARP12. The PC analysis of the existing results confirmed that the FEL analysis is complimented to our previous RMSD finding.

The g_covar module by PCA monitors the conformational changes of C-α mutant and complexes. In this analysis, every single atom’s collective motion along with directions provides a covariance matrix that uses atomic motion in Cartesian coordinate space. There are two types of motion: 1) correlated and 2) anticorrelated motion. The covariance matrix is constituted by two intense colors; red-colored region, which means atoms are moving together, and the blue-colored region, which means atoms are moving in the opposite direction. The matrix depicts that the reduction of negative correlation is largely exhibited with the ZINC03830332 complex. The covariance matrix results demonstrated that all the complexes were tolerable atomic displacement of ZINC03830332, ZINC03830554, and ZINC03831186 (Supplementary Figure S4). The increment in negative correlation was observed in the case of mutant, while reduction of both negative and positive motion was noted in the case of the ZINC03831189 complex. The overall positional fluctuations on Cα-atoms of all the docked complexes revealed that ZINC03830332, ZINC03830554, and ZINC03831186 were stable with steady atomic displacement and amplitude toward PARP12.

The atomic density distribution was measured using mutant PARP12 and complexes ZINC03830332, ZINC03830554, ZINC03831186, and ZINC03831189 ranging from 1.35 nm^−3^, 1.89 nm^−3^, 2.3 nm^−3^, 1.88 nm^−3^, and 1.38 nm^−3^, respectively (Supplementary Figure S5). The results showed the stable density area for ZINC03830332, ZINC03830554, ZINC03831186, and ZINC03831189. The density area of each atom of the complexes ZINC03830332, ZINC03830554, and ZINC03831186 was examined to be in stable distribution; the energy wells displayed three compounds signifying the binding at the same binding site and the stabilized PARP12 enzyme.

## 4 Discussion

Poly [adenosine diphosphate (ADP)-ribose] polymerases (PARPs) are one of the attractive and promising targets to treat several life-threatening diseases including cancer (Kim et al., 2011; Bai and Virág, 2012; Langelier et al., 2018). The present study was undertaken to identify promising lead(s) against the mutated PARP12 enzyme. We carried out an extensive *in silico* study, including virtual screening, drug-likeliness, MD simulations, and MMGBSA analyses. To the best of our knowledge, no computational studies on PARP12 have been carried out before. To this end, virtual screening of the ZINC-FDA dataset of 3,100 compounds was performed.

Molecular docking studies revealed the interaction of the compounds within the active site of receptor via multiple amino acids. For instance, other than H-bonds, the compound ZINC03830332 was also stabilized by Gly564, Thr566, Val570, Gly573, Ile574, Cys575, His577, Phe579, Asp580, Trp581, Arg582, Val583, Cys584, Gly585, Val586, Thr589, Gly594, Ser595, Phe597, Ala598, Asp600, Tyr603, His605, Phe607, Ser659, Ile660, and Phe661 residues. Furthermore, the best four hits (ZINC03830332, ZINC03830554, ZINC03831186, and ZINC03831189) were selected based on their docking score and ADME/T profile used for further studies. The results of the drug-likeness study revealed that the selected compounds obeyed Lipinski’s properties very well and have a high propensity to be developed as a drug.

The MD simulation study revealed that mutation in the enzyme altered the enzyme dynamics as revealed by higher fluctuation throughout the MD simulation. However, upon interaction with the ligand, PARP12 gets stabilized as indicated by smooth fluctuations and showed steady-state throughout the MD simulation. The results of the Rg analysis indicated stable folding behavior of PARP12 after binding with the compounds. The results suggested that the Rg values of complexes with four ZINC-FDA compounds stayed strongly bound to the binding site and maintained stable and enhanced compactness of the PARP12 structure better than the mutant system throughout the MD simulation. The H-bonding profile indicated that compounds ZINC03830332 and ZINC03830554 formed stable complexes throughout the MD simulation. The FEL demonstrated several energy minima corresponding to the metastable conformational states. This analysis observed that the main free energy wells in the global free energy minima area were altered by the ZINC-FDA compounds’ complex bound with PARP12.

In addition, SASA study results showed that all the compounds achieved stable hydrophobic contacts, establishing the maximum region of PARP12 complexes accessible to the solvent molecules. The docked complexes exhibited higher average SASA profiles and stability than the native ligands.

## 5 Conclusion

In the present study, we used pharmacoinformatic techniques to identify potential inhibitors of mutant PARP12 receptors. Summarily, we conducted virtual screening, drug-likeliness, MD simulations, and MMGBSA to identify the best lead compounds amongst the ZINC-FDA compound library. Virtual screening–based molecular docking was carried out to select the best compounds capable of binding to the mutant PARP12 binding site, which helped shortlist the four compounds. Furthermore, Lipinski’s rule of three and ADME/T properties checked the best four compounds with various parameters to get probable drug candidates. The RMSD, RMSF, H-bonds, essential dynamics, and FEL wells analysis dictated that out of 3,100 compounds, only three ZINC-FDA compounds were the most stable, which perfectly bind to the binding pocket PARP12. Overall, the results indicated that the compounds ZINC03830332, ZINC03830554, and ZINC03831186 were better than others. These promising candidates could be used in the future and should be further explored clinically.

## Data Availability

The publicly available datasets were analyzed in this study. These data can be found at: https://zinc.docking.org/.
