# Supplementary material for: Identification of PARP12 Inhibitors By Virtual Screening and Molecular Dynamics Simulations
Source: Front Pharmacol. 2022 Aug 9;13:847499. doi: 10.3389/fphar.2022.847499 (PMC9395932; doi:10.3389/fphar.2022.847499)
Supplement: Supplementary file 1 [file DataSheet1.docx]

Identification of PARP12 Inhibitors using Virtual Screening and Molecular Dynamics Simulations

Tahani M. Almeleebia^1†^, Shahzaib Ahamad^2†^, Irfan Ahmad^3^, Ahmad Alshehri^4,^ Ali G. Alkhathami^3^, Mohammad Y. Alshahrani^3^, Mohammed A. Asiri^3^, Amir Saeed^5,6^, Dharmendra K. Yadav^7^*, Mohd Saeed^8^*

^1^Department of Clinical Pharmacy, College of Pharmacy, King Khalid University, P.O. Box 61413, Abha, 9088, Saudi Arabia.

^2^School of Biotechnology, IFTM University, Lodhipur Rajpoot, Delhi Road, Moradabad, India.

^3^Department of Clinical Laboratory Sciences, College of Applied Medical Sciences, King Khalid University, P.O. Box 61413, Abha, 9088, Saudi Arabia.

^4^College of Applied Medical Sciences, Najran University, P.O. Box 1988, Najran, Saudi Arabia.

^5^Department of Clinical Laboratory Sciences, College of Applied Medical Sciences, University of Hail, Hail, Saudi Arabia.

^6^Department of Medical Microbiology, Faculty of Medical Laboratory Sciences, University of Medical Sciences & Technology, Khartoum, Sudan.

^7^Gachon Institute of Pharmaceutical Sciences and Department of Pharmacy, College of Pharmacy, Gachon University of Medicine and Science, Incheon, 21924. South Korea.

^8^Department of Biology, College of Sciences, University of Hail, Hail, Saudi Arabia.

*Correspondence:

**Prof. A. (Dr.) Mohd Saeed**

Associate Professor

[mo.saeed@uoh.edu.sa](mailto:mo.saeed@uoh.edu.sa)

**Dharmendra K. Yadav, Ph.D.**

Assistant Professor

[dharmendra30oct@gmail.com](mailto:dharmendra30oct@gmail.com)

Tel.: +82-32-820-4948

**Figure SF1.** Chemical structures of four best hits out of 3100 compounds screened.

**Figure SF2.** Representation of binding pocket of PARP12 H-bond interaction with ZINC03830554 displayed in white stick and *PARP12* in yellow colour cartoon model with red stick residues (A), 2D plot of the same complex (B). Compound ZINC03831189 illustrated in green stick and *PARP12* in gold colour cartoon model. (C). 2D plot of ZINC03831189 same complex (D).

**Figure SF3.** 2-D diagram of Hydrogen bond monitoring dynamics of mutant PARP12 and complexes with ZINC03830332 (A), ZINC03831186 (B), ZINC03831189 (C) and ZINC03830554 (D) structures forming hydrogen bonds throughout the total run time of 500 ns each.

 **Figure SF4.** The covariance matrix representation of correlated and anti-correlation motions.

**Figure SF5.** Density distribution of mutant system and complexes with compounds ZINC03830332, ZINC03831186, ZINC03831189 and ZINC03830554 throughout 0-500ns MD simulations.

**Table T1.** ADME/T and RO3 pharmacological properties of ZINC03830332, ZINC03830554, ZINC03831186 and ZINC03831189 compounds.

| **Table 1A. ADME/T properties** | | | | | | | | | | | | | | | | | | | | | | |
| --- | --- | --- | --- | --- | --- | --- | --- | --- | --- | --- | --- | --- | --- | --- | --- | --- | --- | --- | --- | --- | --- | --- |
| **Compound** | | **MW** | | **dipole** | | **SASA** | | **FOSA** | | **FISA** | | **PISA** | | **WPSA** | | **volume** | | **DonorHB** | | **AccptHB** | **RO3** | |
| **ZINC03830332** | | 642.86 | | 8.27 | | 832.50 | | 459.56 | | 371.87 | | 0 | | 1.06 | | 1624.64 | | 13 | | 10 | 1 | |
| **ZINC03830554** | | 693.01 | | 5.00 | | 1083.70 | | 713.96 | | 368.88 | | 0 | | 0.86 | | 2070.61 | | 14 | | 7 | 1 | |
| **ZINC03831186** | | 430.67 | | 1.35 | | 714.08 | | 639.69 | | 74.39 | | 0 | | 0 | | 1387.52 | | 2 | | 5.1 | 1 | |
| **ZINC03831189** | | 422.60 | | 5.26 | | 714.15 | | 540.60 | | 85.31 | | 88.23 | | 0 | | 1359.87 | | 0 | | 4 | 1 | |
| **Table 1B. ADME/T properties** | | | | | | | | | | | | | | | | | | | | | | |
| **Compound** | **MW** | | **dipole** | | **SASA** | | **FOSA** | | **FISA** | | **PISA** | | **WPSA** | | **volume** | | **DonorHB** | | **AccptHB** | | | **RO3** |
| **ZINC03830332** | 642.86 | | 8.27 | | 832.50 | | 459.56 | | 371.87 | | 0 | | 1.06 | | 1624.64 | | 13 | | 10 | | | 1 |
| **ZINC03830554** | 693.01 | | 5.00 | | 1083.70 | | 713.96 | | 368.88 | | 0 | | 0.86 | | 2070.61 | | 14 | | 7 | | | 1 |
| **ZINC03831186** | 430.67 | | 1.35 | | 714.08 | | 639.69 | | 74.39 | | 0 | | 0 | | 1387.52 | | 2 | | 5.1 | | | 1 |
| **ZINC03831189** | 422.60 | | 5.26 | | 714.15 | | 540.60 | | 85.31 | | 88.23 | | 0 | | 1359.87 | | 0 | | 4 | | | 1 |

**Minimal Ranges:** MW = Molecular Weight ( 130.0 / 725.0), accPthB= Acceptor – H-Bonds ( 2.0 / 20.0), rotor=No. of Rotatable Bonds ( 0.0 / 15.0), logP o/w = log P for octanol/water ( -2.0 / 6.5) , dipole=Dipole Moment ( 1.0 / 12.5), logS= log S for aqueous solubility (-6.5 / 0.5), SASA =Total solvent accessible surface area ( 300.0 /1000.0), CIlogS = log S - conformation independent ( -6.5 / 0.5), FOSA =Hydrophobic solvent accessible surface area ( 0.0 / 750.0), logBB= log BB for brain/blood ( -3.0 / 1.2), FISA =Hydrophilic solvent accessible surface area ( 7.0 / 330.0), log Kp = log Kp for skin permeability (Kp in cm/hr), PISA =Carbon Pi solvent accessible surface area ( 0.0 / 450.0), log Khsa = log Khsa Serum Protein Binding ( -1.5 / 1.5), WPSA=Weakly Polar solvent accessible surface area ( 0.0 / 175.0), Lipinski Rule of 3 Violations-RO3 (maximum is 4), % Human Oral Absorption in GI (+-20%) (<25% is poor), volume=Molecular Volume (A^3) (500.0 /2000.0), Apparent Caco-2 Permeability (nm/sec) (<25 poor,>500 great), donorHB= Donor – H-Bonds (0.0 / 6.0), Apparent MDCK Permeability (nm/sec) (<25 poor,>500 great)

.
